# P-138. Effect of diabetes mellitus on the disease entity and clinical parameters in culture positive melioidosis

**DOI:** 10.1093/ofid/ofae631.343

**Published:** 2025-01-29

**Authors:** Bhagya Piyasiri, Pathirawasam Krishantha Jayasekera, Enoka Marie Corea

**Affiliations:** Teaching Hospital Karapitiya, Galle, Southern Province, Sri Lanka; Teaching Hospitak Karapitiya, Galle, Sri Lanka, Galle, Southern Province, Sri Lanka; Faculty of Medicine, University of Colombo, Colombo, Western Province, Sri Lanka

## Abstract

**Background:**

Melioidosis is a multi-system infection caused by *Burkholderia pseudomallei*, a bacterium living in soil which is transmitted to humans through inoculation, inhalation or ingestion. According to numerous studies, diabetes mellitus (DM) is the major risk factor, not only predisposing to melioidosis but also altering the immune response to the infection.

Objectives of this study were; to analyze the prevalence of DM among a local cohort of culture positive melioidosis patients and to assess the effect of DM on the disease entity and clinical parameters.

**Methods:**

Anonymized data of 83 culture positive melioidosis patients from December 2014 to February 2024 were accessed to analyze disease entity, risk factors and laboratory investigations on admission. Fisher’s exact test was used to determine any statistical association between variables and a p value of less than 0.05 was taken as significant.

**Results:**

Among 83 patients, 59 (71.1%) were diabetics. Age groups >60yrs females and < 20yrs males were 100% diabetics. Among 40-60yrs males had 88% prevalence of DM. There were patients with abscesses including deep seated ones (35, 42%), pneumonia (17, 21%), septic arthritis (7, 9%), pyelonephritis (6, 7%) and other disease entities (18, 22%) including rare ones like endocarditis (2, 2%). Mortality rate was 18/83 (22%) of which 13 (72%) were DM.

There was a significant association between abscesses/pus formation and the presence of DM (p=0.0016). Melioidosis antibody titer was tested in 64 (77%) and there was a highly significant association of high titers >1:160 with the presence of DM (p=0.0004). Associations of white cell count (WCC) >11000/µL, and ESR >100mm/1^st^ hr. with DM were statistically significant (p=0.0219 and p=0.0305 respectively). Association of high platelet counts ( >400000/µL) with DM was not statistically significant (p=0.19750).

**Conclusion:**

Diabetes was a significant comorbidity in culture positive Melioidosis. Meticulous investigation for pus formation in melioidosis among diabetics is recommended to ensure proper treatment including pus drainage. Presence of DM is in significant association with higher antibody titres, high ESR and high WCC which may indicate more chronic or extensive disease needing aggressive and prolonged treatment.

**Disclosures:**

**All Authors**: No reported disclosures

